# Antifungal Activity of Capridine β as a Consequence of Its Biotransformation into Metabolite Affecting Yeast Topoisomerase II Activity

**DOI:** 10.3390/pathogens10020189

**Published:** 2021-02-09

**Authors:** Iwona Gabriel, Kamila Rząd, Ewa Paluszkiewicz, Katarzyna Kozłowska-Tylingo

**Affiliations:** Department of Pharmaceutical Technology and Biochemistry, Gdańsk University of Technology, 80-233 Gdańsk, Poland; kamila.rzad@pg.edu.pl (K.R.); ewa.paluszkiewicz@pg.edu.pl (E.P.); katarzyna.kozlowska-tylingo@pg.edu.pl (K.K.-T.)

**Keywords:** *Candida albicans*, acridine, antifungal, topoisomerase, inhibitor

## Abstract

In the last few years, increasing importance is attached to problems caused by fungal pathogens. Current methods of preventing fungal infections remain unsatisfactory. There are several antifungal compounds which are highly effective in some cases, however, they have limitations in usage: Nephrotoxicity and other adverse effects. In addition, the frequent use of available fungistatic drugs promotes drug resistance. Therefore, there is an urgent need for the development of a novel antifungal drug with a different mechanism of action, blocking of the fungal DNA topoisomerases activity appear to be a promising idea. According to previous studies on the m-AMSA moderate inhibitory effect on fungal topoisomerase II, we have decided to study Capridine β (also acridine derivative) antifungal activity, as well as its inhibitory potential on yeast topoisomerase II (yTOPOII). Results indicated that Capridine β antifungal activity depends on the kind of strains analyzed (MICs range 0.5–64 μg mL^−1^) and is related to its biotransformation in the cells. An investigation of metabolite formation, identified as Capridine β reduction product (IE1) by the fungus *Candida albicans* was performed. IE1 exhibited no activity against fungal cells due to an inability to enter the cells. Although no antifungal activity was observed, in contrast to Capridine β, biotransformation metabolite totally inhibited the yTOPOII-mediated relaxation at concentrations lower than detected for m-AMSA. The closely related Capridine β only slightly diminished the catalytic activity of yTOPOII.

## 1. Introduction

**Figure 1:**
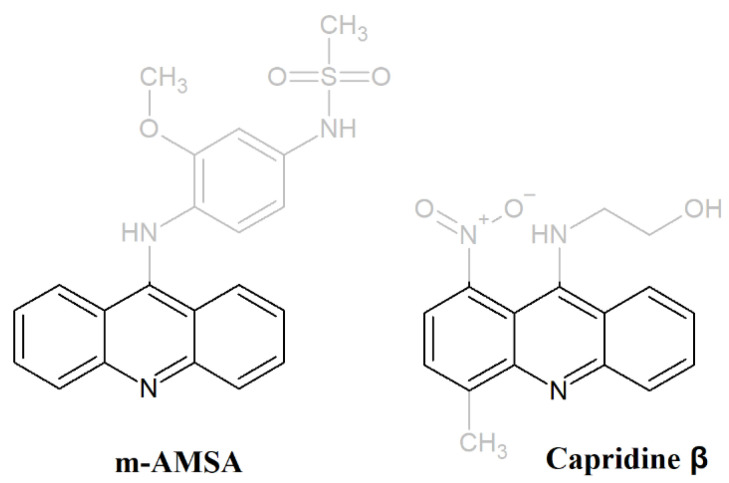
M-AMSA and Capridine β (C-1748) structures with a stressed acridine chromophore.

Acridine derivatives are a class of compounds with a broad spectrum of biological activity and are very interesting for scientists. Many compounds containing the acridine chromophore were synthesized and tested, and the aminoacridines found wide use, both as antibacterial agents and as antimalarials [1]. The planar acridine scaffold, an important pharmacophore, is also a source of new compounds with anti-tumor activity [2]. Surprisingly, although acridine and acridone derivatives are widely analyzed as antibacterial or anticancer agents, only a few reports have demonstrated their antifungal activity [3]. The acridine antimicrobial mode of action, apart from being DNA-targeting drugs, may also be associated with an inhibitory effect on bacterial gyrase or topoisomerase activity. DNA topoisomerases are essential enzymes that catalyze topological changes in DNA. Those enzymes play key roles in replication, transcription, recombination, and chromosome condensation [4]. Camptothecin, its derivatives and novel noncamptothecins target eukaryotic type IB topoisomerases. Human type IIA topoisomerases are the targets of the commonly used anticancer agents etoposide, anthracyclines, and mitoxantrone. Bacterial type II topoisomerases (gyrase and Topo IV) are targeted by quinolones and aminocoumarin antibiotics [5,6]. Functional topoisomerase II is also essential for the growth of yeast [7,8]. The differences between fungal and human topoisomerases have also led to the suggestion that this class of enzymes may become potential targets for the development of novel antifungal agents [9,10]. Moreover, previous studies suggested that fungal and mammalian topoisomerases respond in a different manner to 4′-(9-acridinylamino)methanesulfon-m-anisidide (m-AMSA) (Figure 1), etoposide, and its derivatives A-80198 and A-75272 (a tricyclic quinolone) [11]. Thus probably, there are sufficient biochemical differences between those enzymes to obtain selectivity for fungi over human cells. What is more, all known yeast topo I amino acid sequences contain a characteristic insert absent in the mammalian enzyme [12,13]. The function of this insertion is not known but the development of selective fungal-topo inhibitors is plausible.

1-Nitro-9-aminoacridine derivative Capridine β, 9[2′-hydroxyethylamino]-4-methyl-1-nitroacridine (also known as C-1748) (Figure 1), similar to m-AMSA, exhibited anti-cancer and antifungal properties [14,15,16]. Moreover, previous studies, performed for Capridine β in cancer cell cultures and human xenograft animal models indicated a high therapeutic index and low cytotoxicity with the potential for clinical development [14,15,17,18,19]. Both compounds, m-AMSA, as well as Capridine β, contain an acridine chromophore (Figure 1).

Due to the fact that m-AMSA, a 9-aminoacridine derivative, is a human topoisomerase II poison and has been shown to have a moderate inhibitory effect on fungal topoisomerase II [11] along with a high therapeutic index, low cytotoxicity of Capridine β [14,15,17,18,19], antifungal activity [16], and acridine chromophore similarity to m-AMSA, we have decided to deeply analyze its antifungal potential as well as mechanism of action.

## 2. Results and Discussion

### 2.1. Antifungal Activity

As Capridine β antifungal activity has been previously observed [16], we have decided to analyze the m-AMSA in vitro antifungal activity against five corresponding strains. Minimal inhibitory concentrations (MICs) of the studied compounds determined by the microplate serial dilution method are shown in Table 1.

Results indicated that analyzed acridine derivatives antifungal activity depends on the kind of strains analyzed. Among them, the most active compound was Capridine β. A comparison of *C. albicans* ATCC 10231 growth kinetics in the absence and presence of Capridine β and Amphotericin B at concentrations corresponding to ½ × MIC, 1 × MIC and 2 × MIC indicates its high antifungal efficacy, slightly lower than that of Amphotericin B activity (Figure S1A, Supplementary Materials). As far as m-AMSA is concerned, the analysis of growth kinetics shows that much higher concentrations (32 as well as 64 μg mL^−1^) do not result in a 90% growth inhibition (Figure S1B, Supplementary Materials).

### 2.2. Biotransformation of Capridine β in Fungal Cells

**Figure 2:**
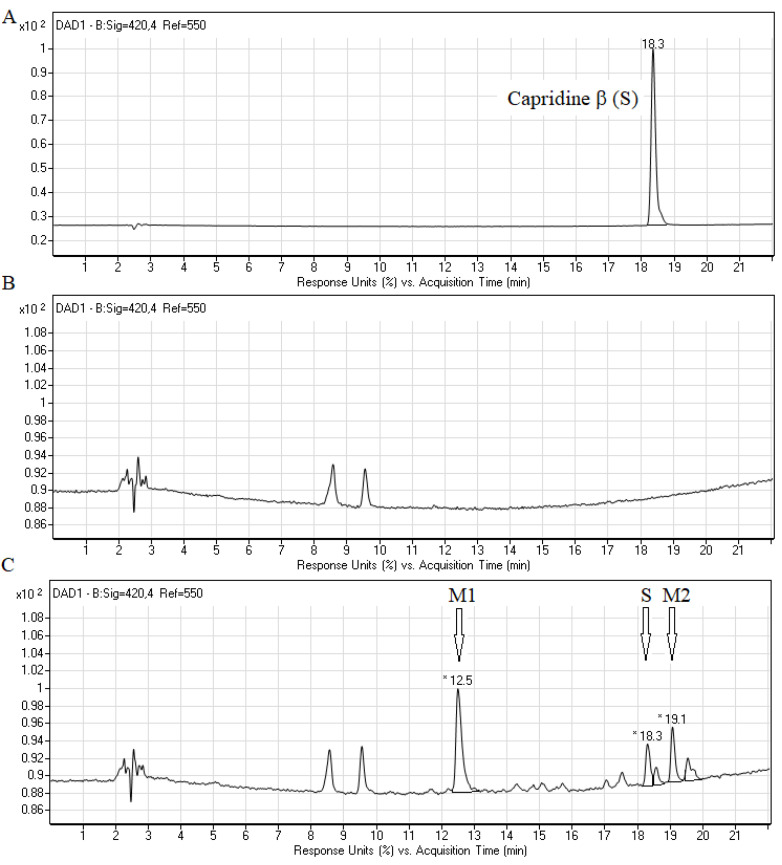
Metabolism of Capridine β (substrate) in *C. albicans* ATCC 10231 cells. RP-HPLC profiles of the 0.1 mM Capridine β (**A**), as well as free cell extracts obtained after incubation of the cells without (**B**) or with (**C**) the studied compound (0.2 mM Capridine β) for 20 h at 30 °C. The cell layer was washed several times and centrifuged, and the final pellet was resuspended in 60% methanol, then disrupted with zirconium-glass beads and centrifuged. The resulting solution was subjected to the RP-HPLC analysis. * Retention times of Capridine β (S) 18.3 min. and metabolites (M1) 12.5 min. and (M2) 19.1 min. are indicated. The symbols of the substrate (S) and its metabolites correspond to those in Figure 3. All the experiments were performed at least in triplicate, and the representative chromatograms are shown.

Due to the significant activity of Capridine β against fungal cells, we have decided to examine whether the original form of the compound or the product of biotransformation is responsible for its activity. Fungal degradation of acridine compounds under an aerobic condition is not well described. Biotransformation experiments were conducted by incubating *C. albicans* cells with Capridine β followed by a small-scale disruption using zirconium-glass beads. Results of high-performance liquid chromatography (HPLC) analyses of the cell free extracts obtained after 20 h of compound (0.2 mM) incubations with *C. albicans* ATCC 10231 are presented in Figure 2. 

The metabolic profile of the studied compound, obtained by HPLC, indicated the presence of three main peaks at retention times (r.t.) 12.5 min (M1), 18.3 and 19.1 min (M2) (Figure 2C). The retention time of 18.3 min was identical to that identified for Capridine β (substrate, Figure 2A). The result indicated that Capridine β was undoubtedly biotransformed into at least two major products M1 and M2.

**Figure 3:**
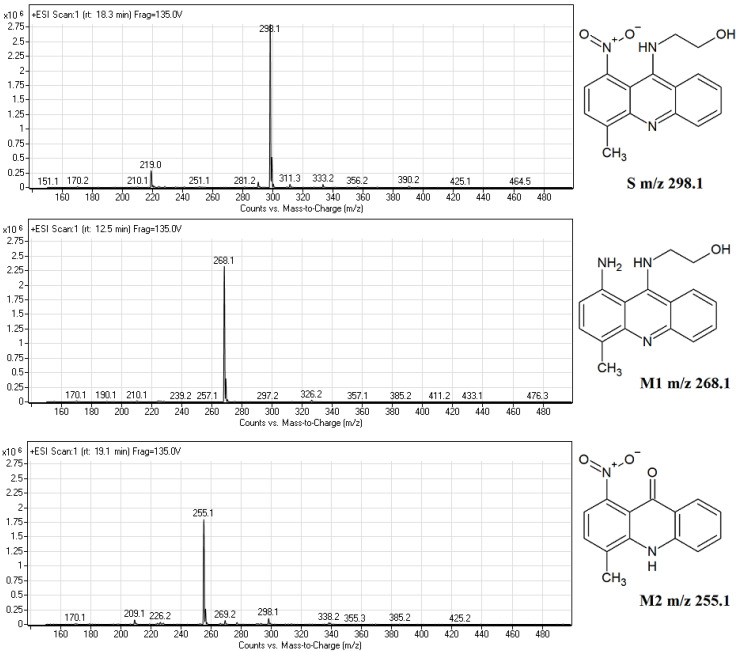
The liquid chromatography mass spectrometry (LC-MS) analysis of three main peaks (r.t. 18.3, 12.5, and 19.1 min) identified as Capridine β (*m*/*z* 298.1) (S) and two main metabolic products M1 (*m*/*z* 268.1) and M2 (*m*/*z* 255.1). All the experiments were performed at least in triplicate, and the representative chromatograms are shown.

Based on the liquid chromatography mass spectrometry (LC-MS) data (Figure 3), two metabolites were predicted to be 1-amino-9-hydroxyethylaminoacridine (M1) and 1-nitroacridinone (M2), mammalian metabolites were also previously reported [15,20].

In contrast to the metabolism of the studied compound in human hepatocellular carcinoma cell lines under normoxia, the metabolites profile observed in *C. albicans* seems to be similar to that obtained for mammalian cells under hypoxia or reducing conditions [15,20]. The main fungal metabolic product M1 (*m*/*z* 268.1), 1-amino-9-hydroxyethylaminoacridine was detected as the main metabolic product of human cancer cell lines only under a low level of oxygen. The second fungal metabolite M2 (*m*/*z* 255.1), 1-nitroacridinone, was previously identified only under reducing conditions with 1,4-dithiothreitol, not found with the studied human enzymatic systems and in HepG2 cells [15]. 

**Figure 4:**
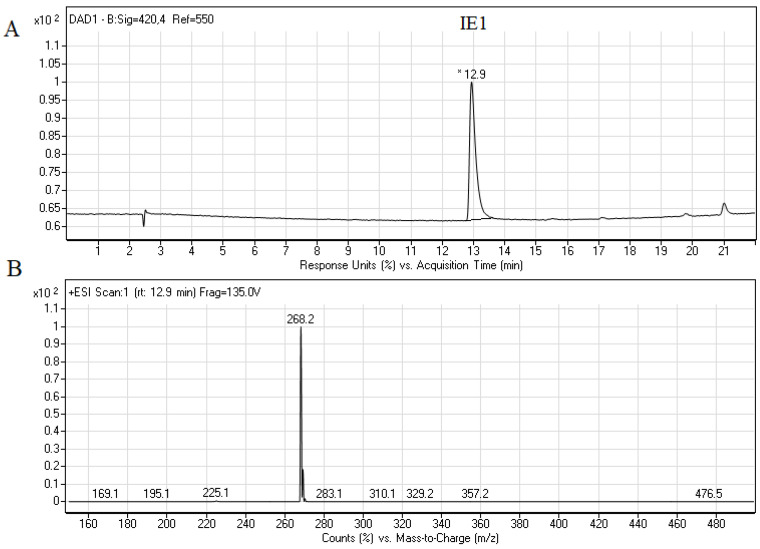
RP-HPLC profiles of the 0.1 mM IE1 (**A**) and its liquid chromatography mass spectrometry (LC-MS) analysis (**B**). * Retention time of IE1 12.9 min. is indicated.

To additionally prove that the main fungal metabolic product M1 was properly identified we have decided to synthesize the reduced Capridine β form (IE1) and perform the RP-HPLC and LC-MS analysis (Figure 4).

The retention time and the molecular ion *m*/*z* 268.2 (Figure 4) found for IE1 were almost identical to those presented for product M1 (Figure 2C and Figure 3). Summing up, we demonstrated that the metabolism of Capridine β in fungal cells differs from that observed for mammalian cells under normoxia and gave two main metabolites, one of them was undoubtedly identified as the reduced form of Capridine β. 

### 2.3. Biological Activity of Capridine β Reduced Form (IE1), Identified as M1 Metabolite

The Capridine β reduced form (IE1), identified as M1 metabolite, was tested for its in vitro antifungal activity against five referenced ATCC strains (Table 1). High MIC values 64 μg mL^−1^ were determined for all the analyzed strains except for *C. albicans* ATCC 10231 (MIC > 64 μg mL^−1^), which indicated small or no antifungal activity. 

**Figure 5:**
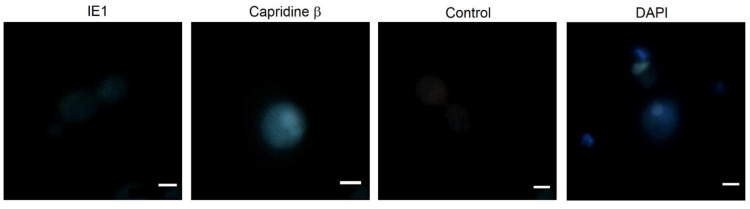
Capridine β and DAPI accumulation in *Candida albicans* ATCC 10231 cells. The cells were incubated in the dark without (control) or with the denoted derivatives (50 μM, 180 min, 37 °C). Compounds fluorescence was observed under a fluorescence microscope (excitation wavelength 400–420 nm, emission >450 nm, ×100). Scale indicated 5 μm.

We next examined the accumulation of the starting compound Capridine β and its reduced form IE1 in the *C. albicans* ATCC 10231 strain using fluorescence microscopy (Figure 5).

**Figure 6:**
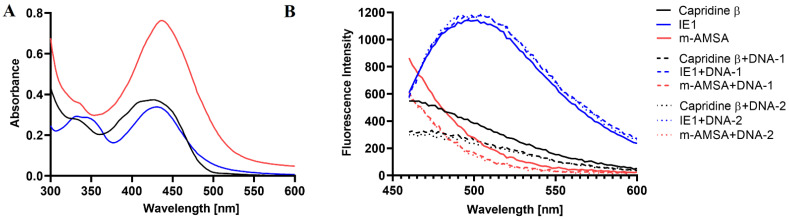
(**A**) UV/vis absorption spectrum of 0.1 mM Capridine β, IE1 or m-AMSA in PBS. (**B**) Background-corrected fluorescence spectra of 0.05 mM Capridine β, IE1, and m-AMSA at the excitation wavelength of 410 nm without (solid line) and with DNA-1 (dashed line) and DNA-2 (dotted line) sequences. Data shown are corrected for background fluorescence and represent averages of at least three replicate samples.

The microscopic analysis revealed that the efficient accumulation of Capridine β in *C. albicans* cells was observed. No accumulation was detected for its reduction product (IE1). Among the analyzed derivatives, fluorescence intensity in the cell did not depend on their own spectral properties in aqueous solutions within the excitation range used for microscopic studies (Figure 6). 

The background-corrected fluorescence spectrum of IE1, obtained in vitro, indicates the highest fluorescence intensity of this compound compared to others (Figure 6) at the excitation wavelength used for microscopic analysis (410 nm) and the same emission range (>450 nm). As it can be seen in Figure 5, the pattern of accumulation for Capridine β seems to be similar to DAPI (4,6-diamidino-2-phenylindole). DAPI staining of *C. albicans* is commonly used to localize the nucleus [21]. Thus, probably the Capridine β targeted structure is a nucleus. According to the previously published studies, an interaction with DNA was observed [14,15,18,22]. Despite the higher IE1 fluorescence intensity, than measured for Capridine β (Figure 6), no fluorescence for that compound was detected within the fungal cells, neither in the cytoplasm nor in the nucleus (Figure 5). Hence, no antifungal activity seems to be the result of an impossibility of reaching the molecular target.

Additional fluorescence studies of Capridine β also revealed its interaction with DNA. In the presence of two different DNA sequences, compound fluorescence quenching was observed (Figure 6). The same effect was observed for m-AMSA. Differently, the presence of DNA enhanced fluorescence intensity of IE1. Thus, probably for that compound a different mode of action for the drug-DNA interaction was observed. As previously reported, when the drugs are bound to DNA, a significant increase in the fluorescence emission is normally observed. In the case of groove binding agents, electrostatic, hydrogen bonding or hydrophobic interactions are involved and the molecules are close to the sugar-phosphate backbone, being possible to observe a decrease in the fluorescence intensity in the presence of the DNA [23].

Acridines and its derivatives are considered to be antimicrobial agents although their activity is obviously determined on the efficient accumulation in bacterial or fungal cells. As described previously for imidazoacridinone C-1311 and its nine derivatives, only three that entered fungal cells showed a phototoxic antifungal activity (C-1330, C-1415, and C-1558) [24]. Despite the high efficiency of C-1311 as an anticancer compound that intercalates into DNA and inhibits human topoisomerase II [25], it was unable to accumulate in *C. albicans* cells and no antifungal activity was observed [24].

**Figure 7:**
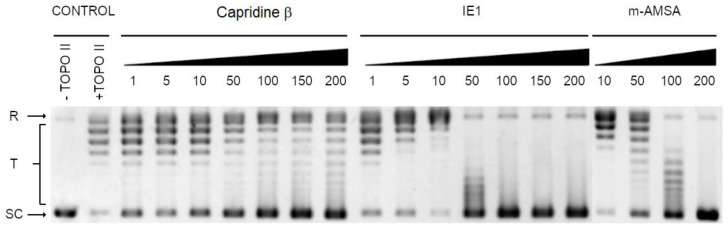
Inhibition of the catalytic activity of purified yeast DNA topoisomerase II by m-AMSA, Capridine β, and IE1 as measured by relaxation. Supercoiled pBR322 plasmid DNA (lane 1) was relaxed by purified yeast topoisomerase II in the absence (lane 2) or presence of m-AMSA at 10, 50, 100 or 200 μM or with analyzed compounds at 1, 5, 10, 50, 100, 150 or 200 μM. The resulting topological forms of DNA were separated by gel electrophoreses. SC: Supercoiled DNA; R: Relaxed DNA; T: DNA topoisomers. Data shown are typical of three independent experiments.

On the basis of the fact that the m-AMSA (aminoacridine derivative) inhibitory effect on fungal topoisomerase II was previously observed [11], we have decided to analyze the influence of Capridine β, as well as its reduced form IE1 on the yeast topoisomerase II (yTOPOII) activity. The relaxation of supercoiled plasmid DNA by yTOPOII was studied in the presence of different concentrations of both compounds (Figure 7).

The most effective IE1 totally inhibited the yeast topoisomerase II-mediated relaxation at concentrations lower than detected for m-AMSA (Figure 7). The inhibition activity of the analyzed compounds was determined by densitometry quantification of the transition from supercoiled to relaxed forms and was expressed in relation to the control. The half maximal effective concentration (EC50) refers to the concentration of a drug, which affected the relaxation in 50%. EC50 determined for IE1 and m-AMSA was 14.1 ± 1.2 μM and >200 μM, respectively. Interestingly, the closely related Capridine β slightly diminished the catalytic activity of topoisomerase II but no complete inhibition was observed in the tested concentration range. 

Acridine and acridone derivatives are widely analyzed as human topoisomerase inhibitors for cancer chemotherapy. M-AMSA in Figure 1 was the first synthetic drug approved for clinical usage that was shown to act as a topoisomerase inhibitor [26]. The molecular mechanism of antitumor triazoloacridinone C-1305 and imidazoacridinone C-1311, both acridine derivatives also indicated its intercalation with DNA as well as the formula of a topo II-stabilizing complex [27,28]. The success of anticancer and antibacterial drugs as DNA topoisomerases inhibitors highlights the potential of topoisomerases from fungal cells as targets for the development of novel antifungals. Fungal topoisomerases might be sufficiently distinct from their human counterparts to enable selective targeting but in order to be active, the antifungal topoisomerase inhibitor needs to enter into fungal cells to reach their intracellular targets. As demonstrated by Kwok et al., no antifungal activity of etoposide was observed [9], although its inhibitory effect on *C. albicans* DNA topoisomerase II was previously reported [11]. 

Results obtained for Capridine β indicate that not only an efficient accumulation, but also the biotransformation into metabolite that can affect fungal topoisomerase II are important with respect to its antifungal activity.

## 3. Materials and Methods

### 3.1. Chemical Synthesis of Capridine β and IE1

#### 3.1.1. General

The products were obtained as the hydrochloride salt. Their structures were confirmed using spectral methods: Mass spectrometry ESI-MS and proton nuclear magnetic resonance (1H NMR). The purity of these compounds was ascertained using thin-layer chromatography (TLC). Melting points were determined on a Stuart SMP30 capillary apparatus. Mass spectra were recorded using an Agilent 6470A triple quadrupole LC/MS system with electrospray ionization source (ESI) in a SCAN mode. Samples were prepared as 1 μg/mL solutions in water and were supplied in 1 μL aliquots to the mass spectrometer in the mixture of acetonitrile:water:formic acid (38:57:5 *v*/*v*/*v*) at a flow rate of 500 μL/min. 1H NMR spectra were recorded on a Varian VXR-S spectrometer operating at 500 MHz. Chemical shifts are reported as δ units in ppm downfield from internal tetramethylsilane. NMR abbreviations used are as follows: m.p.—melting point, br.s—broad signal, s—singlet, d—doublet, t—triplet, m—multiple. The results of the elemental analyses for individual elements fit within ±0.4% of theoretical values.

#### 3.1.2. The Synthesis of Capridine β

9-(2′-Hydroxyethylamino)-4-methyl-1-nitroacridine (Capridine β) was prepared according to the previously reported procedures [29,30]. 

9-(2′-hydroxyethylamino)-4-methyl-1-nitroacridine (Capridine β) 1H NMR (Me_2_SO-d_6_) δ: 8.45 (d, J = 8.3 Hz, 1H), 8.15 (d, J = 8.3 Hz, 1H), 8.08 (d, J = 7.8 Hz, 1H), 7.89 (t, J = 7.8 Hz, 1H), 7.78 (d, J = 7.8 Hz, 1H), 7.54 (t, J = 7.4 Hz, 1H), 3.61–3.75 (m, 4H); ESI-MS [M+H^+^] C_16_H_15_N_3_O_3_—298.2.

#### 3.1.3. The Synthesis of IE1

Capridine β (0.3 mmol) was hydrogenated in the presence of 10% Pd/C (catalytic quantities) in 5 mL methanol by passing gaseous hydrogen through them at room temperature for 24 h. After the time of hydrogenation, the catalyst was filtered off and the solvent evaporated. The product in the form of a base was dissolved in methanol (10 mL) and acidified by the HCl/diethyl ether. After diethyl ether, adding the desired product was obtained. 

9-(2′-Hydroxyethylamino)-1-amino-4-methylacridine (IE1). Yield 92%; m.p. 221–223 °C; 1H NMR (500 MHz, DMSO-d_6_+TFA) δ: 11.35 (s, 1H), 8.31 (d, J = 8.5 Hz, 1H), 8.08 (d, J = 8.5 Hz, 1H), 7.81 (t, J = 7.7 Hz, 1H), 7.40 (d, J = 8.0 Hz, 1H), 7.35 (t, J = 7.7 Hz, 1H), 6.79 (d, J = 7.7 Hz, 1H), 3.95–4.00 (m, 2H), 3.64–3.67 (m, 2H), 2.43 (s, 3H); ESI-MS [M+H^+^] C_16_H_17_N_3_O—268.1. 

### 3.2. Microorganisms Strains and Growth Conditions

The following fungal strains were used: *C. albicans* ATCC 10231, *C. glabrata* ATCC 90030, *C. krusei* ATCC 6258, *C. parapsilosis* ATCC 22019, *S. cerevisiae* ATCC 9763, Fungal strains used in this investigation were routinely grown 18 h at 30 °C in a YPG liquid medium (1% yeast extract, 1% peptone, 2% glucose) in a shaking incubator. For growth on solid media, 1.5% agar was added to the YPG medium.

### 3.3. Metabolism Assays with C. albicans Cells

For studies on cellular metabolism, *Candida albicans* ATCC 10231 strains were grown in the YPG medium overnight (16–18 h) at 30 °C, washed twice with sterile water and resuspended in the fresh YPG medium at a cell density of 2 × 10^8^ cells mL^–1^. In addition, 20 μL of the stock solution of Capridine β in DMSO was added to 980 μL of cell cultures to obtain 0.2 mM final concentrations. The control cells were treated with the same amount of solvent (DMSO). The cells were incubated at 30 °C for 20 h. After incubation, the cells were harvested by centrifugation (4000 rpm, 10 min) and washed with water. Then, the cells were resuspended in 0.5 mL of 60% MeOH at 4 °C and disrupted with the use of zirconium-glass beads (0.5 mm in diameter) by vigorous shaking in four 5-min cycles interrupted by 2-min ice-cooling. The samples were then centrifuged (15 min, 12,000 rpm, 4 °C) and filtered (0.22 μm, PES) prior to the HPLC analysis.

### 3.4. Chromatographic Analysis

The LC-DAD-MS system consisted of a liquid chromatograph, a degasser, a binary pomp, an auto-sampler, and a column oven was combined with a diode array detector (DAD) and MS detector with an electrospray source (AJS ESI) and quadrupole analyzer (1260 Infinity II and 6470 Triple Quad LC/MS, Agilent Technologies, Waldbronn, Germany). The ChemStation software was used to control the LC-MS system and for data processing. The column effluent passed a DAD before arriving in the MS interface.

Chromatographic separations were performed on a Zorbax SB-C18 column (250 mm × 4.6 mm, 5 μm, Agilent Technologies, Santa Clara, CA, USA). For the separation, a gradient of mobile phase A (0.05M HCOONH_4_ in water, pH 7.0) and mobile phase B (100% methanol) was used. The gradient profile was set as follows: 0 min—15% effluent B, 20 min—80% effluent B, 22 min—100% effluent B, 23 min—100% effluent B, 24 min—15% effluent B, 30 min—15% effluent B. The flow rate was 1 mL/min, the column temperature was 25 °C, and the injection volume was 20 μL.

The elution of sample components were monitored at 380 nm, as it has been shown previously for C-1748 and its metabolites in HepG2 cells [15,20] and at 420 nm.

The electrospray source was operated in a positive mode and the interface condition were as follows: Gas temperature 300 °C and flow 5 L/min, sheath gas temperature 250 °C and flow 11 L/min, nebulizer 45 psi, capillary voltage of 3500 V.

The data were collected in a MS scanning mode (MS2 SCAN) with the range 150–700 (*m*/*z*).

### 3.5. Antimicrobial Activity Assay

Antifungal in vitro activity was determined by the modified M27-A3 specified by the CLSI [31]. Wells containing serially diluted examined compounds and compound-free controls were inoculated with 12 h cultures of tested strains to the final concentration of 10^4^ fungi colony-forming units (CFU)/mL. Plates were incubated for 24 h at 37 °C and growth was then quantified by measuring an optical density at 600 nm, using a microplate reader (TECAN Spark 10 M; Tecan Group Ltd., Männedorf, Switzerland). The MIC was defined as the lowest drug concentration in which at least a 90% decrease in turbidity, in comparison to the drug-free control, was observed. The antifungal activity was determined in a RPMI-1640 medium buffered to a pH value of 7.0. The final concentration of the compound solvent (DMSO) did not exceed 2.5% volume of the final suspension in each well, and did not influence the growth of the microorganism.

### 3.6. Acridine Derivatives Accumulation in Microbial Cells

*Candida albicans* ATCC 10231 strains were grown in the YPG medium overnight (16–18 h) at 30 °C, washed twice with a sterile phosphate buffered saline (PBS) and resuspended in RPMI 1640 at a cell density of 2 × 10^6^ cells mL^–1^. The inoculum (500 μL) was added to 500 μL RPMI 1640 with 100 μM of the tested compounds. After 180 min, 200 μL of the cells suspension was washed three times with a sterile phosphate buffered saline (PBS), re-suspended in 25 μL 90% (*v*/*v*) glycerol/10% (*v*/*v*) 1× PBS, and transferred to a microscopic slide. The cells were examined with the Olympus BX-60 fluorescence microscope (excitation wavelength 400–410 nm, emission > 455 nm, ×100) equipped with the Olympus XC50 digital camera and cellSens Dimension imaging software.

### 3.7. Yeast Topoisomerase II Relaxation Assay and Inhibition

The inhibition of yeast Topoisomerase II was analyzed according to the relaxation assay kit from Inspiralis (Inspiralis Ltd., NR4 7GJ, Norwich, UK). Briefly, 500 ng of supercoiled pBR322 DNA, 1 mM ATP, 1–200 μM of the analyzed compound were mixed with a reaction buffer (1 mM Tris.HCl (pH 7.9), 10 mM KCl, 0.5 mM MgCl 2, 0.2% (*v*/*v*) glycerol). The reaction was initiated by the addition of an enzyme, allowed to proceed at 30 °C for 30 min and terminated by the addition of 40% (*w*/*v*) sucrose, 100 mM Tris-HCl pH 8, 10 mM EDTA, 0.5 mg mL^−1^ Bromophenol Blue. A two-step extraction with chloroform:isoamyl alcohol (24:1) and butanol water were made and mixtures were analyzed on the 1% agarose gel in a 1× TAE buffer, 3 h, 4.5 V cm^−1^. The gel was stained in a GelRed 3× staining solution for 30 min and photographed with a Gel Doc XR+ Gel Documentation System (Bio-Rad Laboratories, Inc., 1000 Alfred Nobel Drive, Hercules, CA, USA). The relaxation inhibition effectivity (EC50) of the analyzed compounds was determined by densitometry quantification of the transition from supercoiled to relaxed forms and was expressed in relation to the control.

### 3.8. UV-Vis and Fluorescence Analysis

The UV-visible absorption spectra were recorded using a TECAN Spark 10M (Spark 10M; Tecan Group Ltd., Männedorf, Switzerland) at room temperature with a 1 cm path cell. Compounds at concentrations of 0.1 mM were dissolved in a phosphate-buffered saline (PBS), pH 7.4. Fluorescence spectra were recorded with the use of the TECAN Spark 10M microplate mode. In addition, 100 μL of the analyzed compounds at 0.1 mM concentrations were mixed with 100 μL of MQ water or 0.1 mM DNA sequences: DNA-1 5′→3′: CGATATCG (Tm: 24.0 °C, HPLC grade) and DNA-2 5′→3′: CCCTAGGG (Tm: 28.0 °C, HPLC grade) dissolved in MQ water. The fluorescence spectra of Capridine β, IE1, and m-AMSA with or without DNA were recorded at the excitation wavelength of 410 nm, within the excitation range used for microscopic studies.

## 4. Conclusions

DNA topoisomerases are enzymes that catalyze changes in the spatial structure of DNA and play an important role in replication, transcription, and recombination. Beyond their normal functions, those enzymes are significant molecular targets in antimicrobial and anticancer chemotherapy. Our results indicate that their fungal counterpart may also become a promising antifungal target. The evaluation of biological properties of Capridine β indicated that despite its high antifungal activity, the ability to enter the cell and biotransformation into a product that influences the effectiveness of fungal topoisomerase is crucial for its activity. Summing up, the search for antifungal drug candidates targeting topoisomerases among acridine derivatives is undoubtedly worth continuing. 

## Figures and Tables

**Table 1:** Antifungal activity of Capridine β, IE1, and m-AMSA. * MIC_90_: Minimal inhibitory concentrations at which 90% of cells were inhibited. The experiments were performed at least in five replicates.

Compound	* MIC_90_ μg mL^−1^				
	*Candida albicans* ATCC 10231	*Candida glabrata* ATCC 90030	*Candida krusei* ATCC 6258	*Candida parapsilosis* ATCC 22019	*Saccharomyces cerevisiae* ATCC 9763
Capridine β	1	8	8	64	0.5
IE1	>64	64	64	64	64
m-AMSA	>64	>64	>64	>64	>64
Amphotericin B	0.5	1	1	1	0.5

## Supplementary Materials

The following are available online at https://www.mdpi.com/2076-0817/10/2/189/s1, Figure S1: Growth kinetics of *C. albicans* ATCC 10231 cells in RPMI-1640 medium containing either m-AMSA, Capridine β or Amphotericin B.

## References

[1] Wainwright M. (2001). Acridine-a neglected antibacterial chromophore. J. Antimicrob. Chemother..

[2] Prasher P., Sharma M. (2018). Medicinal chemistry of acridine and its analogues. MedChemComm.

[3] Gabriel I. (2020). “Acridines” as new horizons in antifungal treatment. Molecules.

[4] Champoux J.J. (2001). DNA topoisomerases: Structure, function, and mechanism. Annu. Rev. Biochem..

[5] Li T.-K., Houghton P.J., Desai S.D., Daroui P., Liu A.A., Hars E.S., Ruchelman A.L., Lavoie E.J., Liu L.F. (2003). Characterization of ARC-111 as a novel topoisomerase I-targeting anticancer drug. Cancer Res..

[6] Pommier Y., Leo E., Zhang H., Marchand C. (2010). DNA topoisomerases and their poisoning by anticancer and antibacterial drugs. Chem. Biol..

[7] Dinardo S., Voelkel K., Sternglanz R. (1984). DNA topoisomerase II mutant of *Saccharomyces cerevisiae*: *Topoisomerase* II is required for segregation of daughter molecules at the termination of DNA replication. Proc. Natl. Acad. Sci. USA.

[8] Holm C., Stearns T., Botstein D. (1989). DNA topoisomerase II must act at mitosis to prevent nondisjunction and chromosome breakage. Mol. Cell. Biol..

[9] Kwok S.C., Schelenz S., Wang X., Steverding D. (2010). In Vitro effect of DNA topoisomerase inhibitors on *Candida albicans*. Med. Mycol..

[10] Khan S.I., Nimrod A.C., Mehrpooya M., Nitiss J.L., Walker L.A., Clark A.M. (2002). Antifungal activity of eupolauridine and its action on DNA topoisomerases. Antimicrob. Agents Chemother..

[11] Shen L.L., Baranowski J., Fostel J., Montgomery D.A., Lartey P.A. (1992). DNA topoisomerases from pathogenic fungi: Targets for the discovery of antifungal drugs. Antimicrob. Agents Chemother..

[12] Del Poeta M., Toffaletti D.L., Rude T.H., Dykstra C.C., Heitman J., Perfect J.R. (1999). Topoisomerase I is essential in *Cryp-tococcus* neoformans: Role in pathobiology and as an antifungal target. Genetics.

[13] Jiang W., Gerhold D., Kmiec E.B., Hauser M., Becker J.M., Koltin Y. (1997). The topoisomerase I gene from *Candida albicans*. Microbiology.

[14] Augustin E., Moś-Rompa A., Nowak-Ziatyk D., Konopa J. (2010). Antitumor 1-nitroacridine derivative C-1748, induces apoptosis, necrosis or senescence in human colon carcinoma HCT8 and HT29 cells. Biochem. Pharm..

[15] Wiśniewska A., Niemira M., Jagiełło K., Potęga A., Świst M., Henderson C., Skwarska A., Augustin E., Konopa J., Mazerska Z. (2012). Diminished toxicity of C-1748, 4-methyl-9-hydroxyethylamino-1-nitroacridine, compared with its demethyl analog, C-857, corresponds to its resistance to metabolism in HepG2 cells. Biochem. Pharm..

[16] Rząd K., Paluszkiewicz E., Gabriel I. (2021). A new 1-nitro-9-aminoacridine derivative targeting yeast topoisomerase II able to overcome fluconazole-resistance. Bioorganic Med. Chem. Lett..

[17] Tadi K., Ashok B.T., Chen Y., Banerjee D., Wysocka-Skrzela B., Konopa J., Darzynkiewicz Z., Tiwari R.K. (2007). Pre-clinical evaluation of 1-nitroacridine derived chemotherapeutic agent that has preferential cytotoxic activity towards prostate cancer. Cancer Biol..

[18] Ashok B., Tadi K., Banerjee D., Konopa J., Iatropoulos M., Tiwari R.K. (2006). Pre-clinical toxicology and pathology of 9-(2′-hydroxyethylamino)-4-methyl-1-nitroacridine (C-1748), a novel anti-cancer agent in male Beagle dogs. Life Sci..

[19] Ashok B.T., Tadi K., Garikapaty V.P., Chen Y., Huang Q., Banerjee D., Konopa J., Tiwari R.K. (2007). Preclinical toxicological examination of a putative prostate cancer-specific 4-methyl-1-nitroacridine derivative in rodents. Anti-Cancer Drugs.

[20] Augustin E., Niemira M., Hołownia A., Mazerska Z. (2014). CYP3A4-dependent cellular response does not relate to CYP3A4-catalysed metabolites of C-1748 and C-1305 acridine antitumor agents in HepG2 cells. Cell Biol. Int..

[21] Tan X., Fuchs B.B., Wang Y., Chen W., Yuen G.J., Chen R.B., Jayamani E., Anastassopoulou C., Pukkila-Worley R., Coleman J.J. (2014). The Role of *Candida albicans* SPT20 in filamentation, biofilm formation and pathogenesis. PLoS ONE.

[22] Bartoszek A., Konopa J. (1989). 32P-post-labeling analysis of DNA adduct formation by antitumor drug nitracrine (*Ledakrin*) and other nitroacridines in different biological systems. Biochem. Pharm..

[23] Sirajuddin M., Ali S., Badshah A. (2013). Drug–DNA interactions and their study by UV–Visible, fluorescence spectroscopies and cyclic voltametry. J. Photochem. Photobiol. B Biol..

[24] Taraszkiewicz A., Grinholc M., Bielawski K.P., Kawiak A., Nakonieczna J. (2013). Imidazoacridinone derivatives as efficient sensitizers in photoantimicrobial chemotherapy. Appl. Environ. Microbiol..

[25] Bailly C. (2012). Contemporary challenges in the design of topoisomerase II inhibitors for cancer chemotherapy. Chem. Rev..

[26] Wu C.-C., Li Y.-C., Wang Y.-R., Li T.-K., Chan N.-L. (2013). On the structural basis and design guidelines for type II topoisomerase-targeting anticancer drugs. Nucleic Acids Res..

[27] Lemke K., Wojciechowski M., Laine W., Bailly C., Colson P., Baginski M., Larsen A.K., Skladanowski A. (2005). Induction of unique structural changes in guanine-rich DNA regions by the triazoloacridone C-1305, a topoisomerase II inhibitor with antitumor activities. Nucleic Acids Res..

[28] Mazerska Z., Sowiński P., Konopa J. (2003). Molecular mechanism of the enzymatic oxidation investigated for imidazoacridinone antitumor drug, C-1311. Biochem. Pharm..

[29] Konopa J., Wysocka-Skrzela B., Tiwari R.K. (2003). 9-Alkilamino-1-Nitroacridine Derivatives.

[30] Wysocka-Skrzela B. (1986). Research on tumor-inhibiting compounds: Reactions of 1-nitro-9-aminoacridine derivatives, new antitumor agents, with nucleophiles. Pol. J. Chem..

[31] CLSI (2008). Reference Method for Broth Dilution Antifungal Susceptibility Testing of Yeasts.

