# Supplementary material for: Antifungal Activity of Capridine β as a Consequence of Its Biotransformation into Metabolite Affecting Yeast Topoisomerase II Activity
Source: Pathogens. 2021 Feb 9;10(2):189. doi: 10.3390/pathogens10020189 (PMC7916213; doi:10.3390/pathogens10020189)
Supplement: Supplementary file 1 [file pathogens-10-00189-s001.pdf]

Supplementary data

Antifungal activity of Capridine β as a consequence of its biotransformation into metabolite affecting yeast topoisomerase II activity.

Iwona Gabriel *, Kamila Rząd , Ewa Paluszkiewicz, Katarzyna Kozłowska - Tylingo

Department of Pharmaceutical Technology and Biochemistry, Gdańsk University of Technology, 11/12 Narutowicza Str. , 80-233 Gdańsk, Poland;

* Correspondence: iwogabri@pg.edu.pl; Tel.: +48 583486078; Fax: +48 583471144

Table of contents:

C. albicans **ATCC 10231 growth kinetics in the absence and presence of m-AMSA, Capridine β and Amphotericin B ……………………………………...……………………….….………….2**

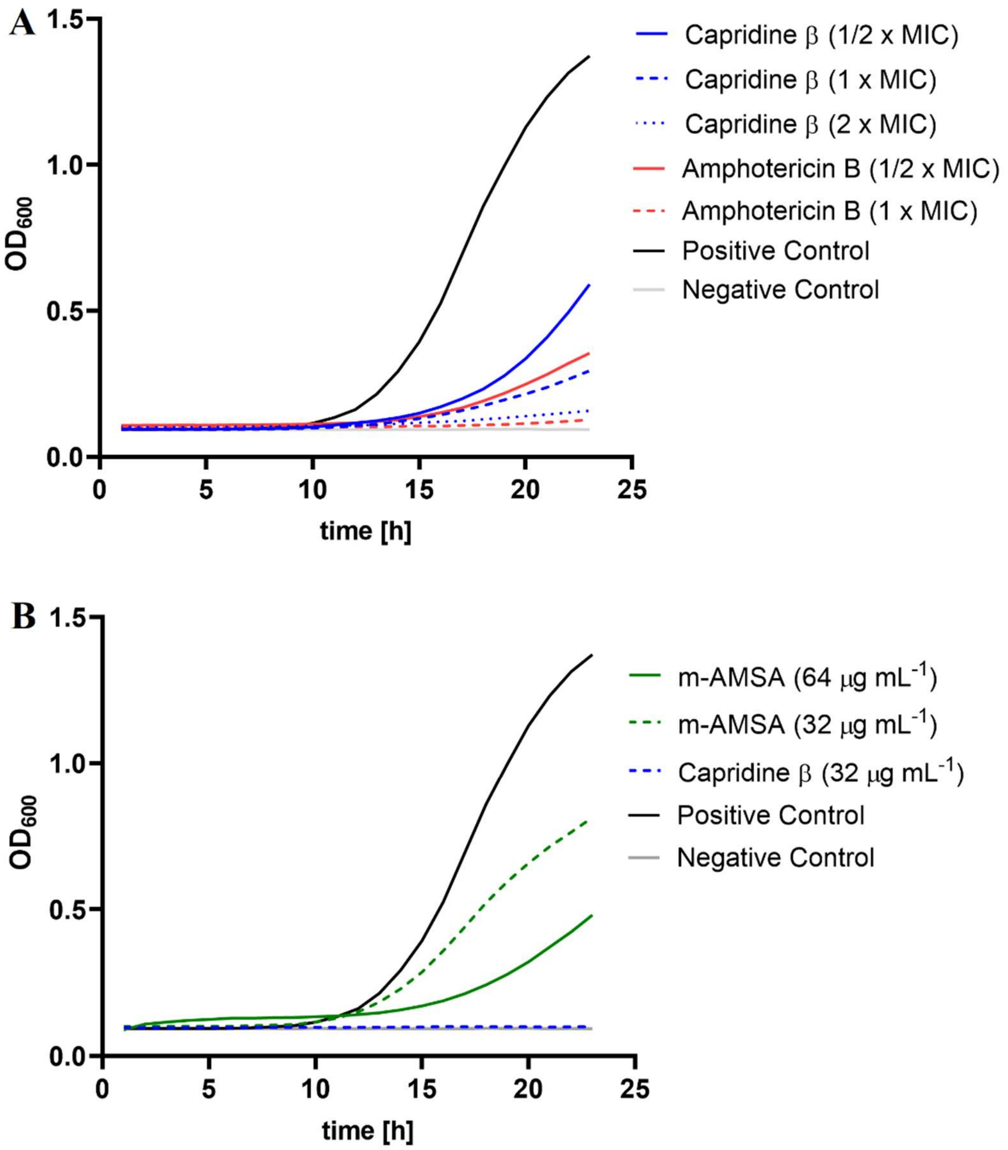

Figure. S1 Growth kinetics of *C. albicans* ATCC 10231 cells in RPMI-1640 medium containing either m-AMSA, Capridine β or Amphotericin B. **A**. Comparison of *C. albicans* ATCC 10231 growth kinetics in the absence (positive control) and presence of Capridine β and Amphotericin B concentrations corresponding to 1/2 x MIC, 1 x MIC, 2 x MIC. **B**. The effect of m-AMSA on *C. albicans* ATCC 10231 growth kinetics. Cell density was measured at time intervals spectrophotometrically (λ = 600nm). Optical density of liquid medium (RPMI-1640) serves as a negative control. All data represent the means ± SD.

Materials and Methods

C. albicans ATCC10231 cells were grown overnight at 30°C in YPG medium. Cells from the overnight culture were washed twice with PBS and suspended to 2×10^4 cells mL^{-1} in RPMI-1640 medium buffered to pH 7.0. Aliquots of 100 µL were used to inoculate the microtiter wells containing 100 µL of RPMI-1640 medium containing tested compounds. Serial 2-fold dilutions of compounds were analyzed starting from 64 μgmL^{-1}. The cell suspensions were cultivated for 24 h at 30°C with shaking and the cell density was measured at time intervals spectrophotometrically (λ= 600 nm) with a microplate reader (TECAN Spark 10M).
